# 环状RNA在非小细胞肺癌放疗抵抗中的研究进展

**DOI:** 10.3779/j.issn.1009-3419.2021.101.37

**Published:** 2021-11-20

**Authors:** 炜龙 李, 胜林 马, 仕蓉 张

**Affiliations:** 1 310053 杭州，浙江中医药大学 Zhejiang Chinese Medical University, Hangzhou 310053, China; 2 310002 杭州，杭州市肿瘤医院 Hangzhou Cancer Hospital, Hangzhou 310002, China; 3 310006 杭州，浙江中医药大学第四临床医学院，杭州市第一人民医院 The Four School of Medicine, Zhejiang Chinese Medical University; Hangzhou First People's Hospital, Hangzhou 310006, China

**Keywords:** 肺肿瘤, CircRNA, 放疗, 抵抗, 研究进展, Lung neoplasms, CircRNA, Radiotherapy, Resistance, Research progress

## Abstract

非小细胞肺癌（non-small cell lung cancer, NSCLC）作为临床上较为常见的癌症，主要特点是患者5年生存率低、预后偏差。目前有部分研究认为生存率低、预后偏差是由于环状RNA（circluar RNA, circRNA）引起了放疗抵抗。因此，找出circRNA与NSCLC放疗抵抗之间的联系，可为改善临床治疗提供理论基础。通过查阅相关文献，找出circRNA与NSCLC放疗抵抗的关系。CircRNA在NSCLC细胞的侵袭、转移、增殖和治疗抵抗中起着重要的作用，其中它所诱导的肿瘤细胞放疗抵抗已经成为放射治疗上的一个重点关注问题。CircRNA在NSCLC的放疗抵抗中起着重要作用。

## Abstract (en)

As the main type of lung cancer, non-small cell lung cancer (NSCLC) is a common cancer which is characterized by low 5-year survival rate and worse prognosis. Nowadays, some studies show that the low survival rate and worse prognosis are due to the resistance to radiotherapy caused by circRNA. Therefore, to find out the relationship between circRNA and radiotherapy resistance of NSCLC was imoprtant. According to research the relevant literatures, the relationship between circRNA and radiotherapy resistance of NSCLC was explored. CircRNA plays an important role in the invasion, metastasis, proliferation and treatment resistance of NSCLC. The radiation resistance of tumor cells induced by circRNA has become a crucial problem in radiotherapy. CircRNA plays an important role in the radiotherapy resistance of NSCLC.

癌症是严重威胁人类生命的一种慢性非传染性疾病，相关研究人员对全球20个主要区域的癌症统计数据^[1]^表明，2012年新增癌症病例1, 410万，死亡病例820万，其中肺癌的死亡率最高，达到160万。而在一项2015年的统计数据^[2]^中显示，我国约有73万的新发肺癌病例，占所有癌症病例的17%，并且仍然有逐年上升趋势，癌症的防治已经成为一个迫在眉睫的问题。然而，在肺癌的主要类型中，非小细胞肺癌（non-small cell lung cancer, NSCLC）约占发病率的85%^[3]^，并且以生存率偏低、预后较差成为肺癌防治中的重点。

如今，随着对癌症的深入认识以及分子生物学的快速发展，科学家通过分子生物学手段对各种生物分子进行详细解析，已经可以揭示部分癌症在发展、侵袭和转移等过程中的相关分子机制。研究^[4-7]^发现，环状RNA（circluar RNA, circRNA）在癌症的进程中发挥了重要的作用，并且与癌症的侵袭、转移、增殖和治疗抵抗等有着密切联系，有可能成为下一代诊断、治疗和疗效预测的新方向。

## 1 CircRNA的生物学结构和作用

### 1.1 CircRNA的生物学结构

早在1976年，科学家在研究高等植物类病毒中发现了circRNA的存在^[8]^，并于1979年用电子显微镜观察到它的结构^[9]^。大多数circRNA是跨物种保守并且稳定的，同时耐RNA酶R，常常表现出组织/发育阶段的特异性表达^[10]^，它是由5'和3'末端以共价键相连组成的线性分子，并拥有不同的长度，既没有5'到3'的极性，也没有poly A尾巴^[11]^。从理论上来说，由于circRNA不具备5'到3'的极性，也没有poly A尾巴，因此认为它没有具备编码的能力，将它归类为非编码RNA家族中的一员。

### 1.2 CircRNA的生物学作用

CircRNA作为一种非编码RNA，在传统的生物学概念上来说它并不具备编码蛋白质的能力，因此也没有太多的生物学特性。然而，随着近几十年来的深入研究，科学家发现它与多种疾病相关，如糖尿病肾病^[12]^、颞叶癫痫^[13]^、心肌纤维化^[14]^和阿尔茨海默病^[15]^等。另外，它还与多种癌症的增殖、侵袭、转移密切相关，近年来发现与癌症相关的circRNA有以下几种（表 1）^[16-33]^。

**表 1:** CircRNA与癌症相关统计 Statistics of the relation between circRNA and cancer

circRNA name	Cancer type	Molecular mechanism	Physiological function
hsa_circRNA_103809	Lung cancer	miR-4302/ZNF121/MYC	Promote progression
circRNA_0000285	Cervical carcinoma	FUS	Promote development
circRNA_101996		miR-8075/TPX2	Promote development
circRNA_PVT1		miR-1286	Promote metastasis
hsa_circRNA_103809	Liver cancer	miR-377-3p/FGFR1/ERK	Promote development
circRNA_10156		miR-149-3p	Promote proliferation
has_circRNA_403658	Bladder cancer	LDHA	Promote growth
circRNA_103809		miR-516a-5p/FBXL18	Promote progression Chemo-resistance
circRNA-000911	Breast cancer	miR-449A	Promote migration and invasion
hsa_circRNA_002178		miR-328-3p	Promote growth
hsa_circRNA_104433	Gastric cancer	miR-497-5p	Promote growth and proliferation
circLPAR3	Esophageal cancer	miR-198	Promote migration and invasion
hsa_circRNA_102209	Colorectal cancer	miR-761/RIN1	Promote growth
circRNA_100146		miR-149/HMGA2	Promote proliferation and invasion
hsa_circRNA_101705	Renal carcinoma	MAPK/ERK	Promote growth and invasion
circRNA_HIPK3	Gallbladder cancer	miR-124	Promote growth
circRNA_0000285	Thyroid cancer	miR-654-3p	Promote proliferation
circ_0074026	Glioma	miR-1304/ERBB4	Promote growth and migration
circRNA: circluar RNA.			

## 2 CircRNA与NSCLC的关系

一系列研究^[34-50]^表明，circRNA与癌细胞的增殖、生长、侵袭等方面存在着密切关系，因此circRNA可以作为生物标志物来诊断、治疗和预测患者预后。目前，已经证明多种circRNA与NSCLC的进展有关，其中部分circRNA与NSCLC的关系如表 2。

**表 2:** CircRNA与NSCLC的关系 Relation between circRNA and NSCLC

Relation with NSCLC	CircRNA name	Molecular mechanism
Progression	circRNA CDR1AS	miR-219a-5p/SOX5
	circVANGL1	miR-195/Bcl-2
	circ_0020123	miR-488-3p
	circZFR	miR-101-3p/CUL4B
	circABCB10	miR-584-5p/E2F5
Prognosis	circPVT1	miR-497
	circ_0003645	miR-1179/TMEM14A
	hsa_circ_000984	Wnt/β-catenin
	circ_0109320	miR-595/ E2F7
Treatment	hsa_circ_0007385	miR-181
	circ_0014130	miR-136-5p/Bcl-2
	hsa_circ_0072309	miR-607
	hsa_circ_0008305	miR-429/miR-200b-3p
	hsa_circ_0102231	miR-145/RBBP4
Diagnosis	hsa_circ_0004050	miR-1233-3p/DUSP9
	circ_0074027	miR-185-3p/BRD4/MADD
	circ_RAD23B	miR-593-3p/CCND2
NSCLC: non-small cell lung cancer.		

## 3 CircRNA与NSCLC放疗抵抗

相关研究^[51-53]^表明，NSCLC与女性乳腺癌、大肠癌和黑色素瘤相比，肺癌的生存率仍然偏低，NSCLC的I期、II期、III期和IV期的5年生存率分别为55.1%、26.2%、15%和4.2%，部分患者还会发生肿瘤局部未控而导致复发，这可能与肿瘤细胞产生了放射耐受性有关。

目前，已有部分研究显示除了长链非编码RNA（long non-coding RNA, lncRNA）^[54]^、微小RNA（micro RNA, miRNA）^[55]^、转录激活因子5（activating transcription factor 5, ATF5）^[56]^、信号传导和转录激活因子3（signal transducer and activator of transcription 3, STAT3）^[57]^、DNA依赖性蛋白激酶（DNA-dependent protein kinase, DNAPKi, M3814）、ATM丝氨酸/苏氨酸激酶（ATM serine/threonine kinase, ATMi）^[58]^和泛素^[59]^等可引起癌细胞的放疗抵抗外，circRNA也可以使细胞获得放射抗性，从而影响局部控制率和患者的生存获益。另外，对细胞circRNA表达谱的研究^[60]^表明，在接受放射后细胞会产生circRNA表达的差异，令细胞产生了放射敏感性的差异。目前，已报道的NSCLC放射抵抗相关的环状RNA，具体有以下几种。

### 3.1 Circ_0086720介导的放疗抵抗

Jin等^[61]^在使用实时荧光定量PCR（quantitative real-time polymerase chain reaction, qRT-PCR）测量circ_0086720、miR-375和Spindlin 1（SPIN1）的表达差异时发现，circ_0086720、miR-375和SPIN1与NSCLC放疗疗效存在一定相关性。在抗放射动物模型的NSCLC组织中circ_0086720和SPIN1的表达增加，而miR-375的表达减少。实验结果发现了circ_0086720通过靶向miR-375并抑制了miR-375的表达，而miR-375与SPIN1结合会抑制SPIN1的表达。后来，通过敲低circ_0086720后发现NSCLC细胞对放射线的敏感性增加，并且可以进一步阻断体内肿瘤的生长，最终证实了circ_0086720的下调是通过调节miR-375/SPIN1通路增强了NSCLC对放射线的敏感性，从而有助于改善NSCLC的放射治疗。

### 3.2 Circ_0001287介导的放疗抵抗

Zhang等^[62]^使用qRT-PCR研究circ_0001287、miR-21表达与NSCLC患者临床病理参数之间的关系发现，circ_0001287在NSCLC组织和细胞系中的表达能力与NSCLC的分化程度和淋巴结转移程度有着密切的关系。Circ_0001287在NSCLC组织和细胞系中的表达均存在下调，引起了癌细胞的放射抗性，而circ_0001287的过表达则可以抑制NSCLC细胞的增殖、迁移、侵袭和放射抗性。更深入的研究显示，circ_0001287可以吸附miR-21并抑制其表达，并间接上调NSCLC细胞中基因功能磷脂酶和张力蛋白同源物（phosphatase and tensin homolog, PTEN）的表达，从而产生上述的效果。

### 3.3 CircMTDH.4介导的放疗抵抗

星形胶质细胞升高基因1（astrocyte elevated gene 1, AEG-1）在多种癌症细胞的侵袭、迁移中发挥着重要的作用，Li等^[63]^收集了28个NSCLC组织和30个正常组织进行NSCLC放疗抵抗研究，并通过定量逆转录-聚合酶链反应和免疫印迹来检测NSCLC细胞中的AEG-1的表达。实验结果表明，circMTDH.4通过miR-630调节AEG-1的表达来使癌细胞获得放射抗性，敲除circMTDH.4或者出现miR-630的过表达均可抑制NSCLC细胞的化学抗性和放射抗性。另外，AEG-1的过表达或miR-630的敲除则发挥了相反的作用。因此，证明了circMTDH.4/miR-630/AEG-1通路与NSCLC细胞的放射抵抗存在一定的关系。

### 3.4 CircRNA ZNF208介导的放疗抵抗

Liu等^[64]^通过分析NSCLC细胞的circRNA差异表达谱发现，circRNA ZNF208在A549细胞和A549-R11细胞中显著上调。在后续的基因敲除实验表明circZNF208可以改变A549和A549-R11细胞对X射线的敏感性。最后，通过一系列的实验证明了circZNF208通过充当miR-7-5p的海绵而上调α-突触核蛋白（α-synuclein, SNCA）的表达，随后促进NSCLC细胞对低线性能量转移（linear energy transfer, LET）X射线的抵抗。另外，这项研究还发现了circRNA ZNF208并不能改变NSCLC细胞对碳离子的敏感性，因此，可以考虑circRNA ZNF208作为一个差异化治疗的靶点。

### 3.5 CircRNA组差异表达介导的放疗抵抗

很多情况下，细胞在接受放射性物质后，会产生DNA链的断裂，从而导致基因突变，最终有一部分细胞会获得对放射性物质的抗性。而这种抗性有可能是通过circRNA的差异性表达来实现的，从而在同一代细胞或下一代细胞间进行传递，使肿瘤细胞获得抗性。

一项关于A549肺癌细胞在接受放射前后的RNA测序的数据^[65]^显示，有1, 875个靶点circRNA在A549细胞中对电离照射发生了差异性的表达。在经过电离辐射处理的A549肺癌细胞中，总共有30个环状RNA表达上调、37个circRNA表达下调。另外，一项关于电离辐射暴露与circRNA表达谱之间关系的研究^[66]^也表明，通过敲除RecQ解旋酶介导的基因组不稳定性蛋白1（recQ-mediated genome instability protein 1, RMI1）可增强辐射敏感性和辐射诱导的凋亡细胞死亡。而在此期间，在已知的RNA库中发现有7, 188个circRNA发生了表达改变，最后分别在敲除RMI1的细胞中没有电离辐射（ionizing radiation, IR）和暴露于电离辐射的细胞中鉴定出有179个和160个差异表达的circRNA。可见，circRNA在细胞中的差异性表达有可能是导致细胞获得放疗抵抗的原因，也将成为一个新的治疗靶点。

## 4 展望与总结

目前，我们虽然拥有化疗、放疗、手术、免疫治疗和靶向治疗等多种与癌细胞对抗的手段，但对于多数NSCLC患者来说，治疗抵抗仍然是治疗效果不理想或病情复发的重要原因。尽管我们通过对癌细胞治疗抵抗机制的深入研究获得了很多关键通路与重要靶点的信息，但是在放疗的过程中会使细胞中的DNA链断裂，从而产生较多的DNA碎片，这样就进一步增加了DNA重组的概率，导致了更多放疗抗性基因的产生，最终降低了放疗的疗效。虽然放疗存在着这些缺点，但为了弥补其他治疗的不足，特别是对一些不能进行手术的NSCLC患者来说，这也是一个重要的治疗方案。

一项III期NSCLC患者生存获益与免疫系统照射剂量相关性的研究^[67]^表明，更高剂量的免疫系统放射线与肿瘤的进展和死亡有关，因此未来的放射治疗方向应该是个体化的、量身定制的。如今，副作用更少的立体定向放射治疗（stereotactic body radiation therapy, SBRT）^[68]^、加速超分割放射治疗（accelerated hyperfractionated radiotherapy, AH-RT）^[69]^和连续加速超分割放射治疗（continuous hyperfractionated accelerated radiotherapy, CHART）^[70]^等新放疗技术已经应用于临床，可以给患者提供更多可选择的放疗方案。因此，放疗在NSCLC患者的全程管理中起到非常重要的作用，而放疗抵抗则很大程度上影响了放疗疗效及患者的生存获益。CircRNA作为一种细胞间传递信息的载体，不但与多种正常的生理活动有关，而且与多种疾病的发生、进展有关，特别是在癌症的增殖、分化、侵袭和浸润等起着重要的桥梁作用，因此进一步研究circRNA在NSCLC放疗抵抗中的分子机制，对于深入了解circRNA介导的放疗抵抗有着重要的作用。目前研究显示circRNA与NSCLC放疗抵抗有着密切的联系，未来可利用circRNA在放疗增敏、放疗敏感和放疗耐受人群中进行筛选以及放疗疗效方面进行预测，为临床优化NSCLC的放射治疗策略提供实验理论基础。
